# The *GBAP1* pseudogene acts as a ceRNA for the glucocerebrosidase gene *GBA* by sponging miR-22-3p

**DOI:** 10.1038/s41598-017-12973-5

**Published:** 2017-10-05

**Authors:** Letizia Straniero, Valeria Rimoldi, Maura Samarani, Stefano Goldwurm, Alessio Di Fonzo, Rejko Krüger, Michela Deleidi, Massimo Aureli, Giulia Soldà, Stefano Duga, Rosanna Asselta

**Affiliations:** 1 Department of Biomedical Sciences, Humanitas University, Via Rita Levi Montalcini, Pieve Emanuele, Milan, 20090 Italy; 2 Humanitas Clinical and Research Center, Via Manzoni 56, Rozzano, Milan, 20089 Italy; 3 Dipartimento di Biotecnologie Mediche e Medicina Traslazionale, Università degli Studi di Milano, Milano, Italy; 4 Parkinson Institute, ASST “Gaetano Pini-CTO”, Milan, Italy; 5 IRCCS Foundation Ca’ Granda Ospedale Maggiore Policlinico, Dino Ferrari Center, Neuroscience Section, Department of Pathophysiology and Transplantation, University of Milan, Milan, Italy; 6 Clinical and Experimental Neuroscience, Luxembourg Center for Systems Biomedicine (LCSB), University of Luxembourg and Centre Hospitalier de Luxembourg (CHL), Esch-sur-Alzette, Luxembourg; 7 German Centre for Neurodegenerative Diseases (DZNE) Tübingen within the Helmholtz Association, Tübingen, Germany; Hertie Institute for Clinical Brain Research, University of Tübingen, Tübingen, Germany

## Abstract

Mutations in the *GBA* gene, encoding lysosomal glucocerebrosidase, represent the major predisposing factor for Parkinson’s disease (PD), and modulation of the glucocerebrosidase activity is an emerging PD therapy. However, little is known about mechanisms regulating *GBA* expression. We explored the existence of a regulatory network involving *GBA*, its expressed pseudogene *GBAP1*, and microRNAs. The high level of sequence identity between *GBA* and *GBAP1* makes the pseudogene a promising competing-endogenous RNA (ceRNA), functioning as a microRNA sponge. After selecting microRNAs potentially targeting both transcripts, we demonstrated that miR-22-3p binds to and down-regulates *GBA* and *GBAP1*, and decreases their endogenous mRNA levels up to 70%. Moreover, over-expression of *GBAP1* 3′-untranslated region was able to sequester miR-22-3p, thus increasing *GBA* mRNA and glucocerebrosidase levels. The characterization of *GBAP1* splicing identified multiple out-of-frame isoforms down-regulated by the nonsense-mediated mRNA decay, suggesting that *GBAP1* levels and, accordingly, its ceRNA effect, are significantly modulated by this degradation process. Using skin-derived induced pluripotent stem cells of PD patients with *GBA* mutations and controls, we observed a significant *GBA* up-regulation during dopaminergic differentiation, paralleled by down-regulation of miR-22-3p. Our results describe the first microRNA controlling *GBA* and suggest that the *GBAP1* non-coding RNA functions as a *GBA* ceRNA.

## Introduction

The glucocerebrosidase gene (*GBA*) encodes for the enzyme glucocerebrosidase (GCase), which catalyzes the hydrolysis of the membrane glucosylceramide (GlcCer) to ceramide and glucose. GCase is mainly a lysosomal enzyme and only partly associated with the outer surface of the cell membrane^1^. GCase deficiency leads to the accumulation of the substrate, responsible for the multi-organ clinical manifestations of Gaucher’s disease (MIM #606463)^2^, one of the most common lysosomal storage disorders^3^. While biallelic mutations in *GBA* are responsible for Gaucher’s disease, heterozygous *GBA* variants have been repeatedly associated with susceptibility to Parkinson’s Disease (PD)^4,5^. Importantly, Gaucher’s and PDs have been connected due to the clinical observation of parkinsonism and Lewy Bodies (LB) pathology in a fraction of patients with Gaucher’s disease^6^. Compared with the general population, patients with the milder form of Gaucher’s disease (type 1) have a 20-fold increased lifetime risk of developing parkinsonism^7^, whereas the odds ratio for any *GBA* mutation in PD patients compared to controls was greater than 5 in a multi-center analysis including more than 5000 cases and 4000 controls^8^. Several studies confirmed that *GBA* mutations, in particular the two most common ones (p.N370S and p.L444P), are more frequent in PD patients than in healthy controls, demonstrating that genetic lesions in this gene are a common risk factor for the disease^9,10^. Recently, we proved the strong relationship between GBA mutations and PD progression and survival^11^.

Despite many efforts, the mechanism underlying the relation between *GBA* mutations and the development of PD remains unclear. There are studies supporting a gain-of-function effect of the mutated protein (promoting α-synuclein aggregation), as well as others supporting a loss-of-function mechanism (leading to substrate accumulation, and hence affecting α-synuclein processing and clearance)^12^. Widespread deficiency of GCase activity has been demonstrated in the brains of PD patients carrying *GBA* mutations, but it is also significant that PD patients without *GBA* mutations were shown to exhibit deficiency of GCase in the *substantia nigra* (SN) as well as in blood^13,14^. Moreover, neurons and brains of PD patients showed accumulation of GlcCer that directly influences the abnormal lysosomal storage of α-synuclein oligomers, thus resulting in a further inhibition of the GCase activity. These findings suggested that the bi-directional effect of GlcCer and α-synuclein accumulation forms a positive feedback loop that may lead to a self-propagating disease^15^. Recent data also linked GCase impairment to the cell-to-cell propagation of α-synuclein aggregates^16^. Based on the above-mentioned evidence, it is plausible that dysregulated *GBA* levels could represent a common feature in PD, whereas loss-of-function *GBA* mutations could constitute the specific trigger responsible for PD development in the *GBA*-associated disease.

Dysregulation of *GBA* expression may, in theory, be due to altered epigenetic, transcriptional, and/or post-transcriptional regulatory mechanisms. In particular, RNA-based networks, characterized by interactions between a specific mRNA, microRNAs (miRNAs), and competitive endogenous RNAs (ceRNAs), are emerging as post-transcriptional regulators of gene expression^17^. Moreover, accumulating evidence points to deregulation of noncoding RNAs as an important and largely unexplored regulatory layer in human neurodegenerative disorders, such as PD^18,19^.

MiRNAs are ~20-nucleotide-long regulatory RNAs that act as post-transcriptional regulators of gene expression by repressing target mRNAs translation and/or by inducing mRNA degradation. About 2000 miRNAs have been experimentally validated in humans and many more have been predicted bioinformatically, making them a major class of regulators^20^. Each miRNA might inhibit the expression of multiple target mRNAs, whose recognition is based on imperfect complementary binding between miRNAs and their target sites, usually located within the 3′ untranslated region (3′UTR)^21^. Recently ceRNAs were described as a novel category of regulatory RNAs: these transcripts compete with mRNAs for miRNAs, acting as molecular “sponges” and thus influencing mRNA levels^17^. Pseudogenes are the best ceRNA candidates, since they have a high-sequence identity with the ancestral gene (and, consequently, they could be targets of the same miRNAs), they can be transcribed, but usually they have lost the ability to generate a functional protein product^22^. Interestingly, a highly-homologous (96% sequence identity) expressed *GBA* pseudogene (*GBAP1*) is located 16 kb downstream of the functional gene^23,24^. *GBAP1* originated from a recent duplication event that occurred no more than 40 million years ago, prior to the divergence of the Great Apes and Old World monkeys, and also involved the metaxin (*MTX*) gene^25,26^.

With the aim to better understand *GBA* expression regulation at the post-transcriptional level, we explored the possible existence of a ceRNA-based network involving *GBA* and *GBAP1*. Here, we demonstrated that *GBAP1* may function as a ceRNA to regulate *GBA* expression by sponging miR-22-3p, thus revealing a novel regulatory circuit that can play a role in the pathogenesis of PD.

## Results

### MiR-22-3p targets *GBA* and *GBAP1*

Since there is no information on miRNAs modulating *GBA* expression, we searched bioinformatically for miRNAs potentially targeting both *GBA* and its pseudogene. Predictions were performed using eight sources of software; candidate miRNA selection was performed by prioritizing miRNAs: i) predicted by at least five algorithms; ii) containing at least 7-nt perfect seed match with *GBA* and *GBAP1* 3′UTRs; iii) known to be expressed in the brain and previously implicated in neurodegenerative diseases. These filtering steps allowed the selection of three candidate miRNAs: miR-22-3p, miR-132, and miR-212. For functional validation, we prioritized miR-22-3p and miR-132, since they were expressed at a higher level in both the cerebellum and frontal cortex (Supplementary Table 1).

**Figure 1:**
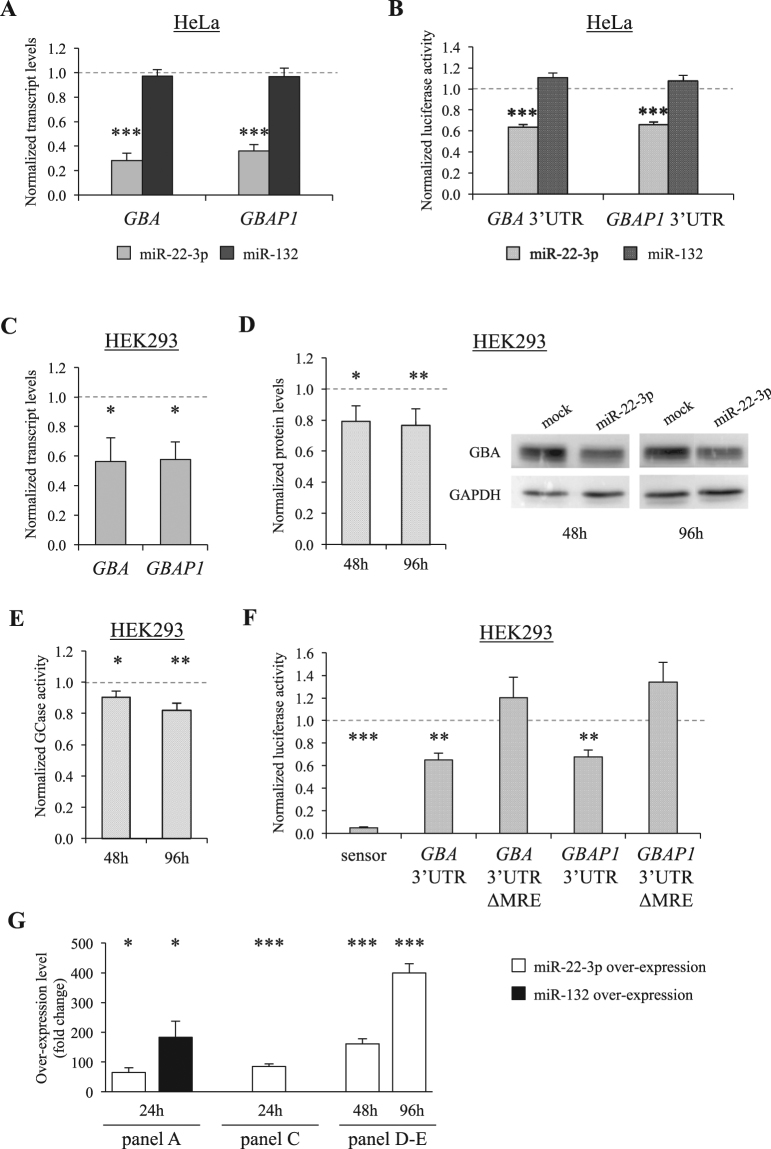
MiR-22-3p targets *GBA* and *GBAP1*. (**A**) Endogenous expression levels of *GBA* and *GBAP1* after pre-miR-22-3p or pre-miR-132 over-expression in HeLa cells. Cells were collected 24 hours after transfection and total RNA extracted. Expression levels, measured by real-time RT-PCRs, are shown as normalized rescaled values, setting as 1 the value measured in cell transfected with an empty vector (psiUX, mock). (**B**) Luciferase reporter assays of *GBA* or the *GBAP1* 3′UTR after pre-miR-22-3p or pre-miR-132 over-expression in HeLa cells. 48 hours after transfection, cells were collected and protein lysates prepared for reporter assays. Renilla luciferase activity was normalized against the firefly luciferase activity, setting as 1 the value measured in cells cotransfected with an empty vector (psiCHECK2, no miRNA overexpression). (**C**–**E**) Effect of pre-miR-22-3p over-expression in HEK293 cells. Panel C shows the effect on the endogenous *GBA* and *GBAP1* transcripts, measured by real-time RT-PCR 24 hours after transfection. Panel D shows the reduction of GBA protein, as assessed by Western blot analysis, 48 or 96 hours after transfection. A representative blot (right) and the densitometric analysis of three independent experiments (left) are shown. Panel E reports the effect on the endogenous GBA-specific GCase activity. In all cases, the value measured in cells cotransfected with an empty vector (psiUX, no miRNA over-expression) was set as 1. (**F**) Luciferase reporter assays of *GBA* or *GBAP1* 3′UTRs, with or without the putative miRNA recognition element (ΔMRE), after miR-22-3p over-expression in HEK293 cells. Cells were collected 48 hours after transfection and lysates prepared for reporter assays. Renilla luciferase activity was normalized against the firefly luciferase activity, setting as 1 the value measured in cells cotransfected with an empty vector (psiCHECK2, no miRNA over-expression). The mir-22-3p sensor^27^ served as positive control. (**G**) MiR-22-3p/miR-132 fold increase reached in each over-expression experiment (detailed below histograms). Error bars represent means +SEM of 3 independent biological replicates, each performed at least in triplicate. In all panels, the reference value, set as 1, is indicated by a dotted line. Significance levels of t-tests are shown. *P < 0.05; **P < 0.01; ***P < 0.005.

To verify that *GBA/GBAP1* can be targets of miR-22-3p and/or miR-132, we cloned both miRNA precursors in a suitable expression vector, and over-expressed them in HeLa cells for 24 hours. The results of these experiments showed that miR-22-3p over-expression can significantly reduce *GBA* and *GBAP1* endogenous mRNA levels (up to 72%; P < 0.0003). Conversely, no *GBA* modulation was detected after miR-132 over-expression (Fig. 1A).

To confirm these results, the 3′UTRs of *GBA* and *GBAP1* were cloned downstream of the luciferase gene in the psiCHECK2 vector. These UTRs differ for only 6 nucleotides, none of them mapping in the predicted binding sites for miR-22-3p and miR-132. We cotransfected in HeLa cells each of these reporter plasmids together with the vector expressing either the miR-22-3p or miR-132 precursor. The results of transfection experiments substantially confirmed previous observations, *i.e*. miR-22-3p was able to target both *GBA* and *GBAP1* UTRs (37% and 34% reduction, respectively; P < 0.0001). Conversely, miR-132 did not affect the expression of the reporter gene (Fig. 1B), and was hence not further investigated.

To better unravel the functional impact of miR-22-3p on the expression of *GBA/GBAP1*, we decided to study miR-22-3p/*GBA/GBAP1* expression profiles in 11 cell lines. Real-time reverse-transcription (RT)-PCRs evidenced a ubiquitous expression of *GBA* in the analyzed lines, with highest levels present in HeLa and glioblastoma cells, and lowest levels in HepG2 cells. *GBAP1* was present in all cell lines, though at lower levels than *GBA* (from 186 to 1.8 times less) (Supplementary Figure 1A). MiR-22-3p showed a nearly ubiquitous expression profile, with highest levels in HepG2 and glioblastoma cells, and lowest levels in HEK293 (Supplementary Figure 1B).

Based on these expression profiles, we decided to repeat miR-22-3p over-expression experiments in HEK293 cells. Results were comparable to those observed in the HeLa cell line, with *GBA* and *GBAP1* endogenous mRNA levels significantly decreased, after 24 hours, up to 44% (P < 0.05; Fig. 1C). We then confirmed the effects of miR-22-3p over-expression also at the protein level. Western blot analysis showed a ~20% reduction in the GBA protein level at both 48 hours and 96 hours after transfection (Fig. 1D). Measurements of endogenous GCase activity demonstrated a 10% and 18% down-regulation after 48 hours and 96 hours of transfection, respectively (P = 0.012 and P < 0.003; Fig. 1E).

Finally, the specific binding of miR-22-3p to *GBA* and *GBAP1* 3′UTRs was demonstrated by deleting the miR-22-3p putative miRNA responsive element (ΔMRE) in the reporter constructs containing the relevant UTR, and subsequently cotransfecting each mutagenized plasmid together with the miR-22-3p expressing one. In these experiments, a luciferase construct containing miR-22-3p antisense sequences (miR-22-3p sensor) was used as a positive control^27^. Our data showed that miR-22-3p responsiveness strictly depends on the presence of the predicted responsive element in the 3′UTR, since its deletion completely abolishes the miRNA-mediated regulation (Fig. 1F). As expected, the level of luciferase activity in the miR-22-3p sensor control dramatically dropped (95% reduction).

### *GBAP1* acts as a ceRNA titrating miR-22-3p and up-regulating *GBA*

We first verified the coexpression of *GBA*/*GBAP1*/miR-22-3p in a broad range of samples (20 human tissues as well as 24 different cerebral regions). The three transcripts were all ubiquitously expressed (Supplementary Figure 2A,B). In particular, *GBA* showed minimal expression in the skeletal muscle and the highest level in the medial temporal cortex (17 fold the skeletal muscle). *GBAP1* expression levels weakly correlated with those of *GBA* (Pearson’s correlation coefficient of 0.53, P < 0.0013, Supplementary Figure 2C), in agreement with the possible ceRNA role of *GBAP1*. Interestingly, *GBAP1* highest expression levels were registered in the brain, where the disproportion between the gene and pseudogene levels is one of the lowest (ratio 1:15). Concerning miR-22-3p, although no consistent anticorrelation was found across the analyzed tissues, the highest miR-22-3p levels were detected in the tissues with lowest *GBA/GBAP1* expression (Supplementary Figure 2).

These results prompted us to verify if altered levels of *GBAP1* could indeed modify the expression of *GBA*. First, as a proof of concept, we over-expressed both the 3′UTR of *GBAP1* and the miR-22-3p hairpin in HEK293 cells. Concurrently, the over-expression experiment was conducted using as sponge the 3′UTR of *GBAP1* without the miR-22-3p responsive element. We showed that *GBAP1* 3′UTR over-expression causes a significant increase in the levels of endogenous *GBA* mRNA only in the presence of the miR-22-3p binding site (1.72 fold; P = 0.019; Supplementary Figure 3A). We also evaluated the ceRNA effect at the protein level, by measuring the GCase activity upon miR-22-3p and *GBAP1* 3′UTR over-expression. Our data confirmed that the *GBAP1* 3′UTR, containing the miR-22-3p binding site, causes a significant increase of GCase activity (1.11 fold; P = 0.013) (Supplementary Figure 3B).

**Figure 2:**
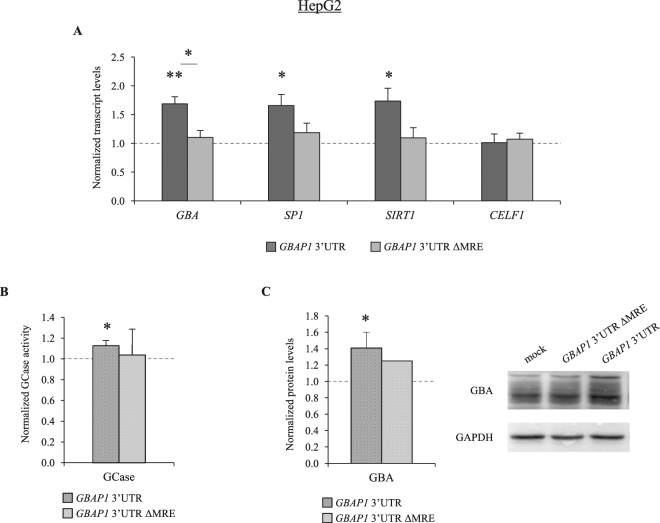
*GBAP1* acts as a ceRNA titrating miR-22-3p and up-regulating *GBA*. (**A**) Effect of *GBAP1* 3′UTR (with or without the miR-22-3p recognition element, ΔMRE) over-expression on the endogenous transcript levels of indicated miR-22-3p targets in HepG2 cells. 24 hours after transfections, cells were collected for extracting total RNA for measurements by semi-quantitative real-time RT-PCRs of: i) *GBA*; ii) *SP1* (Sp1 Transcription Factor; known miR-22-3p target, positive control)^28^; iii) *SIRT1* (Sirtuin 1; known miR-22-3p target, positive control)^29^; and iv) *CELF1* (CUGBP, Elav-Like Family Member 1; negative control). The value measured in cells transfected with an empty vector (psiCHECK2, mock) was set as 1. (**B**) Effect of *GBAP1* 3′UTR (wild type or ΔMRE) over-expression on GCase activity, measured 96 hours after transfections. (**C**) Effect of *GBAP1* 3′UTR (wild type or ΔMRE) over-expression on GBA protein level, measured by Western blot 96 hours after transfections. A representative blot (right) and the densitometric analysis (left) are shown. Error bars represent: means +SEM of 3 (A) or 4 (B) independent biological replicates, each performed at least in triplicate; means +SD of 3 independent biological replicates (C, *GBAP1* 3′UTR). In all panels, the reference value, set as 1, is indicated by a dotted line. Significance levels of t-tests are shown. *P < 0.05; **P < 0.01.

Second, considering the high levels of miR-22-3p measured in HepG2 cells (16-fold the levels measured in HEK293; Supplementary Figure 1), we over-expressed in this cell line the 3′UTR of *GBAP1* alone (with or without the miR-22-3p responsive element) and measured its effect on endogenous *GBA*. We observed a significant increase in the levels of endogenous *GBA* mRNA, once again only in the presence of the miR-22-3p binding site (1.68 fold; P = 0.0016; Fig. 2A). The *GBAP1* ceRNA effect through miR-22-3p sponging was confirmed by measuring the expression levels of known miR-22-3p targets, *i.e*. the *SP1* and *SIRT1* genes^28,29^, which both resulted in up-regulation of ~1.7 fold (P < 0.015). Conversely, no up-regulation was observed for the *CELF1* transcript (Fig. 2A), which does not contain any miR-22-3p responsive element. These results were corroborated by the measurements of GCase activity and GBA protein levels in HepG2 cells under the same experimental conditions (GCase activity: 1.13 fold increase, P = 0.049; GBA protein: 1.40 fold increase, P = 0.020) (Fig. 2B). Finally, a similar overexpression experiment was repeated using as ceRNA the miR-22-3p sensor, which, in principle, represents the “perfect” miRNA sponge. As expected, we observed an up-regulation of all miR-22-3p targets (Supplementary Figure 4).

### The *GBAP1* ceRNA effect could be modulated by the nonsense-mediated mRNA decay (NMD) pathway

To better unravel the reciprocal regulation of the couple *GBA*/*GBAP1*, we decided to comprehensively study *GBA* and *GBAP1* alternative splicing patterns and the possible regulation of expression of these two genes operated by NMD^30,31^.

**Figure 3:**
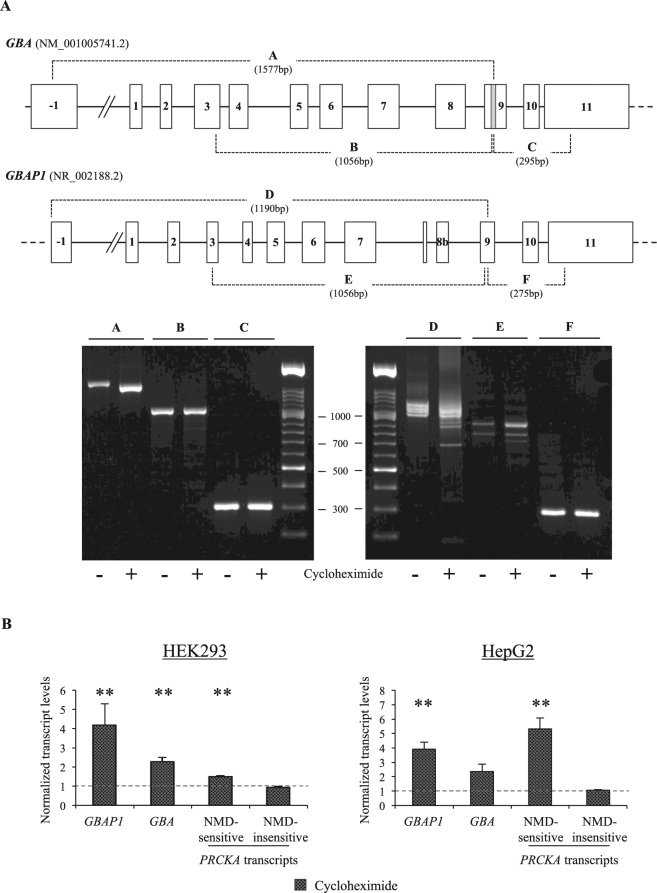
*GBAP1* codes for multiple alternatively-spliced isoforms and is modulated by NMD. (**A**) Analysis of *GBA* and *GBAP1* splicing patterns. In the upper part of the panel, a schematic representation of *GBA* (reference sequence: NM_001005741.2) and *GBAP1* (reference sequence: NR_002188.2) genes is reported. Exons are indicated by boxes, introns by lines. The 55-bp-long sequence characterizing *GBA* exon 9 is specified by a grey rectangle. The scheme is approximately to scale. The overlapping fragments amplified by RT-PCRs to analyze the *GBA* and *GBAP1* splicing patterns are indicated by dashed lines and a letter. In the lower part of the panel, the electrophoretic analysis (agarose gels 2%) of RT-PCR amplicons is shown. RT-PCRs were performed on RNA extracted from HepG2 cells treated (+) or untreated (−) with the NMD inhibitor cycloheximide. On the top of each gel, letters indicate the relevant RT-PCR amplicons. (**B**) Demonstration of the NMD-mediated degradation of *GBAP1* transcripts. The two panel shows expression levels of *GBAP1* and *GBA* isoforms in HEK293 and HepG2 cells, untreated or treated for 8 hours with cycloheximide. Expression levels of endogenous *GBAP1*/*GBA* isoforms were measured by semi-quantitative real-time RT-PCRs. Results are presented as normalized rescaled values, setting as 1 the value of the untreated samples (dotted line). The expression level of the Connexin 43 or 32 transcripts, known to be insensitive to NMD, were used in the normalization step. RT-PCRs performed on out-of-frame and in-frame *PRKCA* isoforms, known to be respectively sensitive and insensitive to the NMD blockage^32^, represent the positive and negative control. Error bars represent means +SEM of 3 independent biological replicates, each performed at least in triplicate. Significance levels of t-tests are shown. *P < 0.05; **P < 0.01.

To capture the vast majority of all possible splicing events, long-range RT-PCR assays were designed to completely cover both genes (Fig. 3A). The specific amplification of either *GBA* or *GBAP1* in each assay was assured by anchoring one primer to exon 9, in correspondence of the pseudogene-specific 55-bp deletion. RT-PCR assays were performed on RNA extracted from HepG2 cells treated or not with the NMD inhibitor cycloheximide. This analysis allowed the identification of multiple alternatively-spliced isoforms for *GBAP1*; conversely, *GBA* did not show any detectable alternative isoform (Fig. 3A). Notably, the heterogeneity of the splicing pattern of *GBAP1* increased after cycloheximide treatment, suggesting that multiple pseudogene splicing isoforms may be modulated by NMD. A tentative reconstruction of the main splicing variants of *GBAP1* was performed by a combination of isoform-specific semi-nested RT-PCRs and DNA sequencing, highlighting the presence of multiple transcripts containing a premature termination codon (Supplementary Figure 5).

The global effect of NMD degradation on *GBA* and *GBAP1* levels was also investigated by semi-quantitative real-time RT-PCR. This analysis showed a significant increase in the expression level of *GBAP1* in treated cells (4.18 and 3.92 fold in HEK293 and HepG2 cells, P = 0.045 and P = 0.0034, respectively), confirming that this pseudogene is down-regulated by NMD (Fig. 3B). Also *GBA* transcripts were up-regulated upon NMD inhibition (2.28 and 2.35 fold in HEK293 and HepG2 cells), a rather unexpected result given the lack of out-of-frame *GBA* isoforms in our preliminary analysis. However, these results suit the hypothesis that *GBAP1* levels may influence *GBA* expression through a ceRNA effect. As control, in-frame and out-of-frame *PRKCA* isoforms, known to be insensitive/sensitive to the NMD blockage^32^, were also analyzed and yielded the expected results (Fig. 3B).

### *GBA*, *GBAP1*, and miR-22-3p are expressed in induced pluripotent stem cells (iPSCs)-derived neuronal cells

To be relevant for the molecular pathogenesis of PD, the *GBA*/*GBAP1*/miR-22-3p network should work in tissues affected by the disease process, *e.g*. dopaminergic (DA) neurons. We thus verified the expression of *GBA*, *GBAP1*, and miR-22-3p in iPSCs and iPSC-derived neuronal cells (after 35 days of differentiation). Semi-quantitative real-time RT-PCR assays were performed on total RNA extracted from iPSCs/neurons derived from fibroblasts of six healthy controls and four PD patients (all carrying *GBA* mutations).

**Figure 4:**
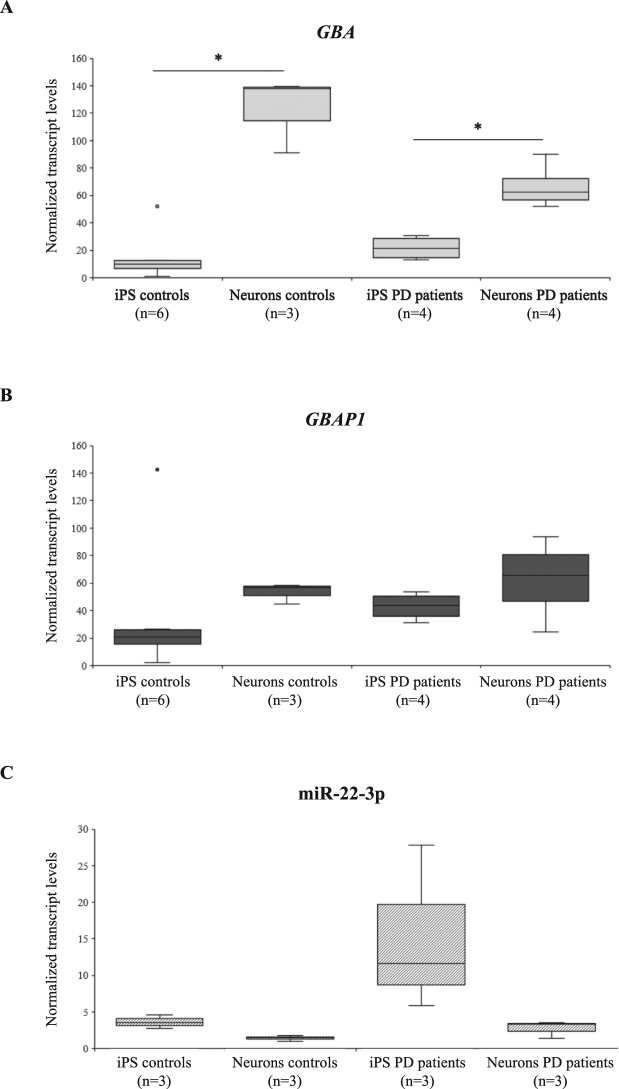
*GBA*, *GBP1*, and miR-22-3p are expressed in iPS cells and iPSC-derived neurons of PD patients and controls. *GBA* (**A**), *GBAP1* (**B**), and miR-22-3p (**C**) expression levels were measured by semi-quantitative real-time RT-PCRs in up to six iPS and iPSC-derived neuronal cells of cases and controls. Boxplots show expression levels according to the disease status; boxes define the interquartile range; the thick line refers to the median. Results are presented as normalized rescaled values. Significance level for differences between groups was calculated by a Wilcoxon-Mann-Whitney test, and showed only if significant. *P < 0.05.

All three players of the regulatory circuit were expressed both in iPSCs and iPSC-derived neurons, respectively. The process of differentiation towards neurons is accompanied by a significant up-regulation of *GBA* (8 fold in controls, P = 0.024; 3 fold in patients, P = 0.029) and by a parallel increase in expression levels of *GBAP1* (Fig. 4A and B). In addition, we detected a trend for down-regulation of the *GBA* transcript in PD patients with respect to controls in DA neurons (0.54 fold, P = 0.057). Finally, consistent with the observed up-regulation of *GBA*/*GBAP1* during neuronal differentiation, we detected lower expression levels of miR-22-3p in DA neurons with respect to their precursors (0.39 fold in controls; 0.18 fold in patients) (Fig. 4C).

## Discussion

**Figure 5:**
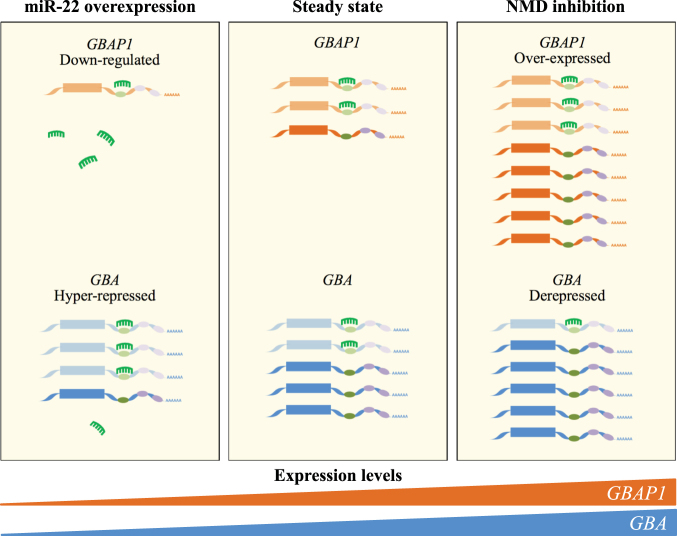
Schematic representation of the effect of modulating the *GBA*/*GBAP1*/miR-22-3p RNA-based network on endogenous *GBAP1* and *GBA* levels. Schematic representation of the ceRNA network involving *GBA* (blue transcripts) and *GBAP1* (orange transcripts), harboring the same MRE sites (green and violet ovals). The green MRE sites bind to miR-22-3p (in green), whereas violet ones bind to other not-specified miRNAs. The experimental modulation of the proposed ceRNA network impacts on both coregulated transcripts. In particular, over-expression of miR-22-3p (left part of the figure) determines the down-regulation of both *GBA* and *GBAP1* transcripts. Conversely, over-expression of *GBAP1* (*e.g*., by inhibiting the NMD pathway, as experimentally verified in the present study; right part of the figure) will increase the cellular concentrations of miR-22-3p MREs, thus resulting in the de-repression of *GBA*. In the scheme, transcripts destined to degradation are colored in lighter shades.

Despite substantial efforts over the past few years to understand the role of long non-coding RNAs (lncRNAs) in health and disease, only some of them have been investigated for their biological function^33^. One promising, although debated, idea assigning to lncRNAs a generalized function is the “ceRNA hypothesis”, based on the fact that specific RNAs can limit miRNA activity through sequestration, thus up-regulating the expression of miRNA target genes^17^. In particular, two classes of lncRNAs are increasingly recognized as main ceRNA contributors, *i.e*. circular RNAs and pseudogene-derived transcripts^34^. Indeed, transcribed pseudogenes, mostly deriving from duplication events, are considered optimal ceRNA candidates, as they share miRNA-binding sites with the ancestral genes^17,34^. To date, a number of pseudogenes have been experimentally demonstrated to act as ceRNAs, including: *PTENP1* and *KRAS1P* ^22^, *OCT4-pg4* ^35^, *BRAFP1* ^36^, and *CYP4Z2P* ^37^. In this study, we describe a novel ceRNA-based network involving *GBA*, its pseudogene *GBAP1*, and miR-22-3p (Fig. 5).

The molecular evolution, expression pattern, and mechanisms of transcriptional regulation of *GBA* have been previously investigated^38–40^, mainly because of its direct link with Gaucher’s disease. On the other hand, the few data available on *GBA* post-transcriptional regulation principally stem from a screening aimed to identify miRNAs regulating the GCase activity in p.N370S homozygous Gaucher fibroblasts^41^. This screening involved 875 miRNAs and evidenced at least three candidates (miR-127-5p, miR-16-5p, and miR-195-5p), exhibiting a Z-score of at least +/−2, with substantial consequences on the GCase activity. However, in all cases, the miRNA effect did not seem to be mediated by a direct binding of the miRNA to *GBA* transcripts; rather, miRNAs acted either on the LIMP-2 receptor, which is involved in the trafficking of GCase from the endoplasmic reticulum to the lysosome, or on the expression levels of known modifiers of the GCase activity^41^. Hence, our work identifies miR-22-3p as the first miRNA directly targeting *GBA*. Interestingly, in the publicly-available dataset of Siebert and coworkers^41^ miR-22-3p mimic resulted to down-regulate GCase activity (Z-score = −1.5; suggestive P = 0.066), according to our results.

Concerning *GBAP1*, sparse information is available to date, and it is primarily focused on the evolution of the *GBAP1 locus* as an example of a very recently acquired pseudogene^23,38^. We hence extensively studied *GBAP1* splicing pattern and expression profile, showing that it is subjected to multiple physiologic in-frame and out-of-frame splicing events and that it is broadly expressed, though often at low levels (Supplementary Figure 2). More interestingly, we showed that *GBAP1* is targeted by NMD, which seems to be the main mechanism regulating its expression level: blocking NMD, the ratio between *GBA* and *GBAP1* substantially increased (on average from 1/100 to 1/68 in HepG2 cells, and from 1/9 to 1/4 in HEK293 cells). Of course, the *GBAP1* expression control exerted through NMD raises the question about the pseudogene translation, since RNAs should undergo a pioneer round of translation, associated with an inspection operated by the NMD machinery, before being degraded^29^. Interestingly, it has been recently reported that many lncRNAs, and even 5′UTRs, are translated, and *GBAP1* was identified among non-canonical human translated open reading frames^42^. The remarkable degradation of *GBAP1* operated by NMD not only may cause its low abundance, but also results in an increased degradation of bound miRNAs, possibly enhancing *GBAP1* efficiency as miRNA sponge, as suggested for other pseudogenes^17^.

The relevance of post-transcriptional regulation in determining the low *GBAP1* expression is also suggested by the observation that *GBAP1* proximal and distal promoters show high level of sequence identity with those of *GBA*, and are hence predicted to have similar transcriptional strength. For instance, the presence of two TATA boxes and two CAAT boxes in the proximal promoter of *GBAP1* exactly recapitulates the architecture of *in-cis* regulatory elements characterizing the *GBA* proximal promoter^23^. Moreover, epigenetic marks are not substantially different when comparing the gene and the pseudogene promoters, as inferred from the UCSC Genome Browser ENCODE tracks (http://genome.ucsc.edu/; release Feb. 2009, GRCh37/hg19). Indeed, our in-house preliminary data, obtained with reporter constructs, show that the activity of *GBAP1* promoters does reach the transcriptional levels of the corresponding *GBA* promoters (data not shown).

Our overexpression experiments in different cell lines clearly demonstrated that *GBAP1* 3′UTR, at supraphysiological concentrations, can modulate *GBA* mRNA levels through a miR-22-3p-mediated regulatory circuit. However, these results do not necessarily imply that this ceRNA-based regulation may also work in more physiological conditions. To confirm that *GBAP1* can act as a *GBA* ceRNA without overexpression, we exploited the predicted differential sensitivity to NMD of the gene and pseudogene transcripts (see Fig. 3A). Cycloheximide treatment allowed us to increase the relative abundance of the endogenous *GBAP1* mRNA of around 4 times the basal level and was accompanied by a 2-fold increase in *GBA* transcripts, not directly attributable to NMD, and compatible with a ceRNA effect (Fig. 3B). Hence, in specific cells or developmental stages, up-regulation of *GBAP1*, resulting from post-transcriptional or epigenetic regulatory mechanisms, might titrate miRNAs away from the *GBA* protein-coding transcripts, thus providing a physiologic ceRNA effect.

The existence of an RNA-based network controlling *GBA* expression suggests the intriguing possibility that miR-22-3p or *GBAP1* dysregulation could also be associated with PD. In this frame, we investigated *GBA*/*GBAP1*/miR-22-3p expression pattern in disease-relevant tissues using *in-silico* analyses of microarray datasets publicly available through the Gene Expression Omnibus repository (see Supplementary Materials and Methods), as well as *in-vivo* measurements performed on RNA extracted from iPSCs and iPSC-derived neuronal cells of PD cases and controls. In particular, we retrieved three microarray datasets evaluating differential gene expression in the SN of *post-mortem* brains, for a total of 51 cases and 42 controls (Supplementary Table 2). In the meta-analysis, we measured a significant down-regulation of both *GBA* and *GBAP1* transcripts in PD patients (P < 0.05; Supplementary Figure 6). Notably, we observed the same significant down-regulation for *GBA* transcripts in iPS-derived DA neurons of PD patients; accordingly, miR-22-3p was slightly, although not significantly, up-regulated in cases vs. controls (on average 1.96 fold, P = 0.13; Fig. 4).

A few studies have reported a potential neuroprotective effect of miR-22-3p in rat models of cerebral ischemia-reperfusion injury, as well as in Huntington’s and Alzheimer’s disease, through a reduction in inflammation and apoptosis^43,44^. However, other studies suggested a pro-senescence role of miR-22 in endothelial progenitor cells, in cancer, and in the aging heart and brain^28,45–47^. While the neuroprotective effects of miR-22 have suggested enhancing its expression as a potential therapeutic strategy for the treatment of neurodegenerative conditions, it may well be that miR-22 overexpression represents a pathophysiologic response to protect the cell from injury and stress also triggering other non-beneficial effects, like increased aging and reduced GCase activity.

In conclusion, we are aware of the fact that the connection between the RNA-based network and PD pathogenesis presented here has not been formally proven. However, one can easily imagine a link between the down-regulation of the sister transcripts *GBA*/*GBAP1* - or, conversely, the up-regulation of miR-22-3p - and an aberrant α-synuclein metabolism, as already theorized^12^. A confirmed dysregulation of the *GBA*/*GBAP1*/miR-22-3p circuit in PD patients would suggest possible novel therapeutic strategies, based either on the direct control of the expression of the miRNA/pseudogene, or on the modulation of the NMD pathway aimed at up-regulating *GBAP1* levels^48^.

## Methods

### Plasmid constructs

MiR-22-3p and miR-132 precursors were inserted into the psiUX expression vector (kindly provided by Prof. I. Bozzoni, Università di Roma La Sapienza, Rome, Italy). *GBA* and *GBAP1* 3′UTRs were directionally cloned downstream of the renilla luciferase gene in the psiCHECK2 reporter plasmid (Promega, Madison, USA). All constructs were produced by PCR amplifying the relevant genomic region from the DNA of a healthy subject using an appropriate PCR primer couple (Supplementary Table 3), and subsequently by cutting the amplified products with the proper restriction enzyme. Restricted products were ligated into the relevant plasmid.

The constructs carrying the *GBA* and *GBAP1* 3′UTR deleted of the miR-22-3p binding site (ΔMRE) were obtained by site-directed mutagenesis, by means of the QuikChange kit (Agilent Technologies, Santa Clara, USA), following the manufacturer protocol.

A pGL3-control luciferase construct containing a single perfectly-complementary miR-22-3p antisense sequence (miR-22-3p sensor), kindly provided by Dr. Da-Zhi Wang (Children's Hospital Boston and Harvard Medical School), was used as a positive control^27^.

All plasmids were purified using the PureYield™ Plasmid Miniprep System kit (Promega) according to the manufacturer’s instructions. All recombinant and mutagenized vectors were verified by conventional Sanger sequencing, as described^10^.

### Prediction of *GBA/GBAP1*-targeting miRNAs

Predictions were performed using publicly-available algorithms: microRNA.org^49^, MicroCosm Targets^50^, PITA^51^, as well as the miRWalk2 suite^52^.

### Cell cultures and transfection experiments

HEK293, HepG2, and HeLa cells (kind gift of Prof. D. Fornasari and Prof. A. Rollier, University of Milan, Milan, Italy) were cultured according to the standard procedures. All cell lines were routinely tested for mycoplasma contamination.

For miRNA over-expression experiments, cells were cotransfected using 3.5 µg (HeLa) or 875 ng (HEK293) of the psiUX plasmid expressing either miR-22-3p or miR-132 precursors.

For the miRNA-target interaction analysis, HEK293 cells were cotransfected using 300 ng of the psiUX plasmid expressing miR-22-3p together with 720 ng of the psiCHECK2 plasmid containing the relevant 3′UTR.

For the ceRNA-effect analysis, HEK293 cells were cotransfected using 300 ng of the psiUX plasmid expressing miR-22-3p together with 300 ng of the psiCHECK2 plasmid containing the *GBAP1* 3′UTR. HepG2 cells were transfected with 300 ng of the *GBAP1* 3′UTR only or with 300 ng of the miR-22-3p sensor (as positive “sponge” control).

In each experiment, an equal number of cells (2.5 * 10^5^ for HeLa, 3 * 10^5^ for HEK293, 4 * 10^5^ for HepG2) were transfected with the Polyplus jetPRIME (EuroClone, Wetherby, UK) in 6-well plates, as described by the manufacturer. Depending on the measurement to be performed at the end of experiment, cells were collected 24, 48, 72, or 96 hours after transfection (detailed in the relevant figure legend), to obtain either total RNA, or cell lysates (see below).

### RNA samples

Expression profiles of *GBA*, *GBAP1*, and miR-22-3p were determined using RNA from: a panel of 20 human tissues (First Choice total RNA; Ambion, Austin, USA), a panel of 24 human cerebral regions (Clontech Laboratories, Palo Alto, USA), 11 cell lines, iPSCs, and DA neurons differentiated from iPSCs (see below).

RNA from cell lines, iPSCs, DA neurons, as well as transfected cells was isolated using the Eurozol kit (Euroclone), according to the manufacturer’s protocol. RNA concentration/quality was assessed using the Nanodrop ND-1000 (Thermo Fisher Scientific, Waltham, USA).

### Semi-quantitative real-time RT-PCR

For the evaluation of expression levels of specific genes, random hexamers and the Superscript-III Reverse Transcriptase (Invitrogen, Carlsbad, USA) were used to perform first-strand cDNA synthesis starting from 1 µg of RNA extracted from cells, or RNA derived from a panel of human tissues. From a total of 20 µL of the RT reaction, 1 µL was used as template for amplifications using the FastStart SYBR Green Master Mix (Roche, Basel, Switzerland) on a LightCycler 480 (Roche), following a touchdown thermal protocol. Expression levels were normalized using *HMBS* (hydroxymethylbilane synthase gene) and *ACTB* (β-actin) as housekeeping genes. To discriminate between the quasi-identical *GBA* and *GBAP1* genes, we took advantage of the 55-bp deletion in exon 9 characterizing *GBAP1* as well as of the few nucleotide differences between *GBA* and *GBAP1* spread along the two genes.

MiR-22-3p and miR-132 levels were measured by real-time RT-PCR by a poly(A) tailing and a universal reverse transcription approach, using the miRNA First Strand Synthesis kit (Agilent Technologies) and starting from 300 ng of total RNA, according to the manufacturer’s instructions. RT-PCR reactions were performed using the universal reverse primer (Agilent Technologies) and miRNA-specific forward primers, as described^53^. U6 snRNA was used as housekeeping gene. Real-time reactions were performed as described above.

In all cases, real-time RT-PCR assays were performed at least in triplicate on a LightCycler 480, and expression levels were analyzed by the GeNorm software^54^. Correlation between *GBA*/*GBAP1*/miR-22-3p expression profiles was calculated using the Pearson’s correlation. Pearson’s coefficients <−0.5 and >0.5 are considered as anti-correlation and positive correlation, respectively. P values <0.05 were considered as statistically significant.

Primer couples used in RT-PCR assays are listed in Supplementary Table 3.

### Luciferase assays

For miRNA-target interaction assays, the activities of firefly/renilla luciferase were measured in lysates from transfected cells by using the Dual-Luciferase Reporter Assay System (Promega) and the Wallac 1420 VICTOR^3^ V reader (PerkinElmer, Waltham, USA). The values of renilla luciferase were normalized against the corresponding values of firefly luciferase.

### Western blot analysis

Cells were lysed in water containing protease and phosphatase inhibitors (Sigma-Aldrich, Saint Louis, USA) on ice using an ultrasonic homogenizer. Total cell protein content was measured using the DC Protein Assay (Bio-Rad, Hercules, USA). In total, 40–50 μg of the protein lysate was loaded on a 10% polyacrylamide gel and transferred on a PVDF membrane (GE Healthcare, Freiburg, Germany). Blots were incubated with primary antibodies overnight at 4 °C on a shaker platform (Anti-GBA ab128879 1:2.500, Abcam, Cambridge, USA; anti-GAPDH G9545 1:7.000, Sigma) and were then probed with anti-rabbit IgG-HRP secondary antibody (1:2.000, Cell Signaling, Leiden, The Netherlands) for 1 h at room temperature. Visualization was done by using Westar ETA C 2.0 ECL Substrate for Western Blotting (Cyanagen, Bologna, Italy). For quantitative measurements, membranes were acquired using the Uvitec Cambridge technology (Eppendorf, Hamburg, Germany). Image analysis was performed with the Uvitec software.

### GCase enzymatic activity assays

Cells to be assayed were washed twice with phosphate buffered saline (PBS), harvested, and then lysed in water containing complete protease inhibitor cocktails (Roche). Total cell protein content was measured using the Micro BCA assay reagent (Pierce, Rockford, USA). Cells lysates were transferred to a 96-well microplate and assays were performed in triplicate. Cell-lysate associated GCase activity was analyzed using 4-methylumbelliferyl- β-D-glucopyranoside (MUB-Glc; Glycosynth, Warrington, UK), solubilized at a final concentration of 6 mM in McIlvaine Buffer (0.1 M Citrate/0.2 M Phosphate, pH 5.2) containing 0.1% Triton X-100. As Triton is a selective inhibitor of β-glucosidase2 (*GBA2*) activity, these conditions allowed the specific measurement of GBA-related GCase activity^55,56^. The reaction mixtures were incubated at 37°C under gentle shaking. The fluorescence was recorded after transferring 10 μL of the mixture in the microplate and adding 190 μL of 0.25 M glycine, pH 10.7. The fluorescence was detected by a Wallac 1420 VICTOR^3^ V reader. Data were expressed as pmoles of converted substrate/mg cell proteins × hour.

### *GBA* and *GBAP1* splicing pattern and sensitivity to the NMD pathway

Analysis of *GBA*/*GBAP1* splicing patterns and susceptibility to NMD was undertaken in HepG2 and HEK293 cell lines. Cells were plated at a density of 4 * 10^5^ per 6-well dish and, after 72 hours, treated for 8 hours with cycloheximide (100 µg/mL; dissolved in dimethyl sulfoxide) or with the vehicle alone. After the treatment, cells were washed with PBS and total RNA extracted.

For the analysis of the splicing pattern, a set of gene-specific or pseudogene-specific RT-PCR assays (Supplementary Table 3) was designed to catch the vast majority of possible alternative splicing events. RT-PCRs were performed as described above. The main amplified products, recovered from the agarose gel using the Wizard SV Gel and PCR Clean-Up System kit (Promega), were directly sequenced to confirm their identity.

Variations in the expression levels of *GBA*/*GBAP1* upon treatment were quantified by real-time RT-PCR assays using as reference an NMD-resistant transcript (*i.e*., Connexin 43 or Connexin 32 mRNAs, whose coding sequences are all contained in a single exon, for HEK293 and HepG2, respectively). The NMD-sensitive and insensitive *PRKCA* transcripts were used as controls^32^.

### Fibroblast-derived iPSCs

IPSC lines derived from skin fibroblasts of six controls and four PD patients carrying heterozygous *GBA* mutations (p.L444P, n = 2; p.N370S, n = 2) were previously described^57^ and were obtained following the protocol of Takahashi and colleagues^58^. These iPSCs were subjected to neuronal differentiation for 35 days *in vitro*, according to Kriks and collaborators’ protocol^59^.

This study has the approval of the local Ethics Committees (Parkinson Institute, ASST “Gaetano Pini-CTO”, Milan, Italy; IRCCS Foundation Ca’ Granda Ospedale Maggiore Policlinico, Milan, Italy; Medical Faculty and the University Hospital Tübingen, Tübingen, Germany) and was performed according to the Declaration of Helsinki. Signed informed consent was obtained from all participants.

### Data availability

The authors declare that the data supporting the findings of this study are available within the article and its Supplementary material file or from the corresponding authors on request.

## Electronic supplementary material

Supplementary Information
