# Supplementary material for: The GBAP1 pseudogene acts as a ceRNA for the glucocerebrosidase gene GBA by sponging miR-22-3p
Source: Sci Rep. 2017 Oct 5;7:12702. doi: 10.1038/s41598-017-12973-5 (PMC5629250; doi:10.1038/s41598-017-12973-5)
Supplement: Supplementary file 1 — Supplementary Information [file 41598_2017_12973_MOESM1_ESM.doc]

**SUPPLEMENTARY INFORMATION**

**The *GBAP1* pseudogene acts as a ceRNA for the glucocerebrosidase gene *GBA* by sponging miR-22-3p**

Letizia Straniero,^1^ Valeria Rimoldi,^2^ Maura Samarani,^3^ Stefano Goldwurm,^4^ Alessio Di Fonzo,^5^ Rejko Krüger,^6^ Michela Deleidi,^7^ Massimo Aureli,^3^ Giulia Soldà,^1,2,*^ Stefano Duga,^1,2^ Rosanna Asselta^1,2^

^1^ Department of Biomedical Sciences, Humanitas University, Via Manzoni 113, 20089 Rozzano, Milan, Italy

^2^ Humanitas Clinical and Research Center, Via Manzoni 56, 20089 Rozzano, Milan, Italy

^3^ Dipartimento di Biotecnologie Mediche e Medicina Traslazionale, Università degli Studi di Milano, Milano, Italia

^4^ Parkinson Institute, ASST “Gaetano Pini-CTO”, Milan, Italy

^5^ IRCCS Foundation Ca' Granda Ospedale Maggiore Policlinico, Dino Ferrari Center, Neuroscience Section, Department of Pathophysiology and Transplantation, University of Milan, Milan, Italy

^6^ Clinical and Experimental Neuroscience, Luxembourg Center for Systems Biomedicine (LCSB), University of Luxembourg and Centre Hospitalier de Luxembourg (CHL), Luxembourg

^7^ German Centre for Neurodegenerative Diseases (DZNE) Tübingen within the Helmholtz Association, Tübingen, Germany; Hertie Institute for Clinical Brain Research, University of Tübingen, Tübingen, Germany

**List of contents:**

- Supplementary Materials and Methods
- Supplementary figure 1
- Supplementary figure 2
- Supplementary figure 3
- Supplementary figure 4
- Supplementary figure 5
- Supplementary figure 6
- Supplementary table 1
- Supplementary table 2
- Supplementary table 3
- Supplementary references

**SUPPLEMENTARY MATERIALS AND METHODS**

**Microarray dataset retrieving and analysis**

Microarray datasets were retrieved from the National Center for Biotechnology Information (NCBI) GEO database (http://www.ncbi.nlm.nih.gov/gds/). The database was searched using “Parkinson’s disease”, “*substantia nigra*”, and “human” as keywords (accession date: June 14th, 2016). The manual review of the obtained entries allowed the identification of three datasets (GSE7621, GSE8397, and GSE20292), all characterized by expression data obtained from RNAs extracted from the *substantia nigra* of Parkinson’s disease cases and healthy controls. Characteristics of selected datasets are listed in Supplementary Table 3. The three datasets were analyzed separately by comparing Parkinson’s disease cases *vs* healthy controls, using the GEO2R web application (Barrett *et al.*, 2009; Barrett *et al.*, 2013). GEO2R, available at GEO repository, implements Bioconductor R packages (Gentleman *et al.*, 2004; R Core Team, 2013) and provides results as a list of genes ordered by significance (P value). We specifically searched this list for probe sets corresponding to *GBA* and *GBAP1*. The three identified probes (210589_s_at, 209093_s_at, and 216400_at; shared by the three datasets) were then individually blatted against the human genome, using the UCSC Genome Browser (hg19) to verify if they target correctly the corresponding gene. The 216400_at probe, which resulted to recognize intronic sequences, was excluded from further analysis. We hence performed a meta-analysis using the expression data associated with the two surviving probe sets and the Integrative Meta-analysis of Expression Data (INMEX) program (Xia *et al.*, 2013). Data were uploaded to INMEX, processed, annotated, checked, and meta-analyzed by using the “Combining P-values” option, based on the Fisher’s method [-2*∑Log(p)] (Xia *et al.*, 2013). The threshold for significance was set to 0.05.

**
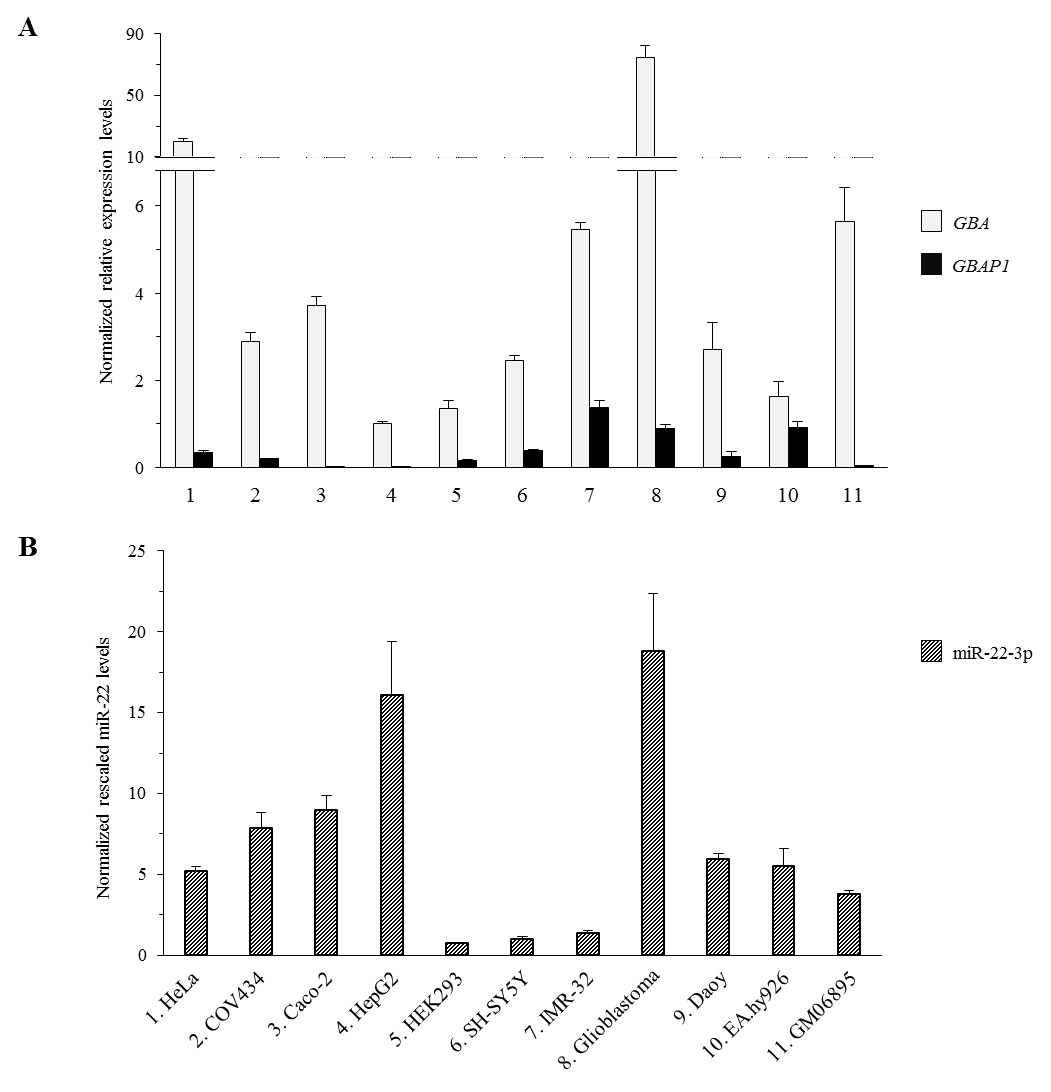
**

**Supplementary figure 1: Expression profiles of *GBA*, *GBAP1*, and miR-22-3p in cell lines.**

**A)** Expression levels of *GBA* and *GBAP1* were measured by semi-quantitative real-time RT-PCRs in a panel of 11 cell lines. *HMBS* and *ACTB* were used as housekeeping genes.

**B)** MiR-22-3p expression levels were determined in the same panel of cell lines using a poly(A) tailing and a universal reverse transcription approach, followed by real-time RT-PCRs. U6 was used as housekeeping gene.

In all cases, expression levels are shown as normalized rescaled values, setting as 1 the value measured in HepG2 (panel A) and SH-SY5Y (panel B). Error bars represent means +SD of three technical replicates.

Cell lines: HeLa (epithelial cervical carcinoma); COV434 (granulosa carcinoma); Caco-2 (epithelial colorectal adenocarcinoma); HepG2 (liver carcinoma); HEK293 (embryonic kidney); SH-SY5Y (neuroblastoma); IMR-32 (neuroblastoma); Glioblastoma; Daoy (medulloblastoma); EAhy296 (endothelium);- GM06895 (lymphoblastoid line).


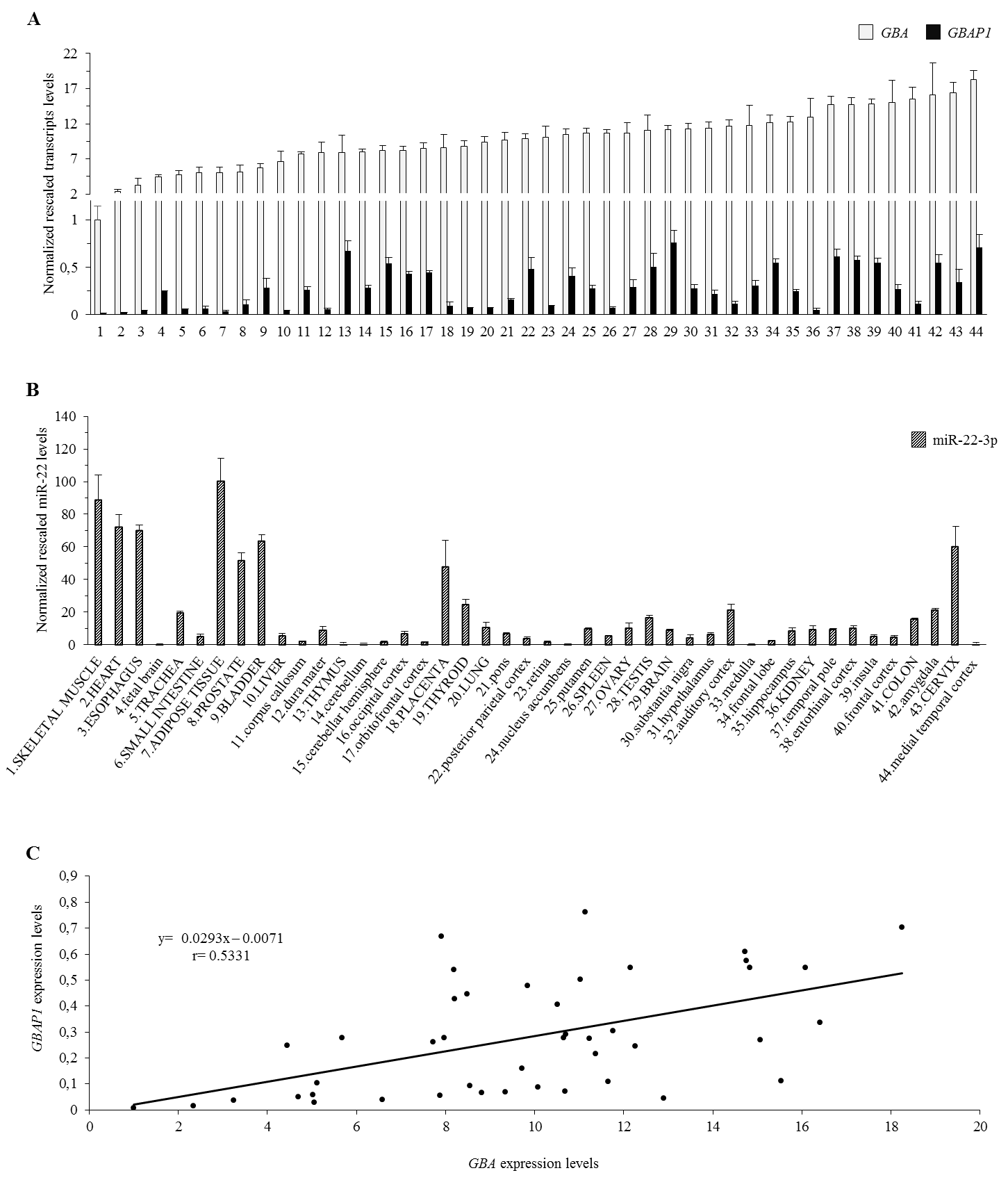


**Supplementary figure 2: Expression profiles of *GBA*, *GBAP1*, and miR-22-3p in 20 human tissues and 24 human cerebral districts.**

Expression levels of *GBA*, *GBAP1*, (**panel** **A**) and miR-22-3p (**panel** **B**) were measured by real-time RT-PCR assays on cDNAs derived from commercial panels of 20 human tissues (listed in upper-case letters) and 24 different RNAs from cerebral districts (in lower-case letters; for more details, see legend of Supplementary figure 1).

Correlation between *GBA* and *GBAP1* expression levels across the 44 analyzed tissues (**panel C**).

Each tissue sample consisted in a pool comprising RNA from at least 3 donors.

Concerning specifications for brain district samples:

- dura mater, parietal cortex posterior, auditory cortex, putamen, frontal cortex, entorhinal cortex, amygdala, hippocampus, orbital frontal cortex, temporal cortex medial, hypothalamus, and pons RNA samples all derive from a single person;

- fetal brain: this sample derives from normal brains of 59 spontaneously aborted male and female Caucasian fetuses (20-33 weeks);

- frontal lobe: pooled from 4 male and female Caucasian individuals (32-61 years old);

- nucleus accumbens: pooled from 6 male and female Caucasian individuals (23-56 years old);

- retina: pooled from 25 male and female Caucasian individuals (24-65 years old);

- insula: pooled from 15 male and female Caucasian individuals (20-68 years old);

- substantia nigra: pooled from 21 male and female Caucasian individuals (20-62 years old);

- medulla oblongata: pooled from 29 male and female Caucasian individuals (18-64 years old).

Results are presented as normalized rescaled values, setting as 1 the values corresponding to skeletal muscle (**panel A**), and thymus (**panel B**). Error bars represent means +SD of three technical replicates.


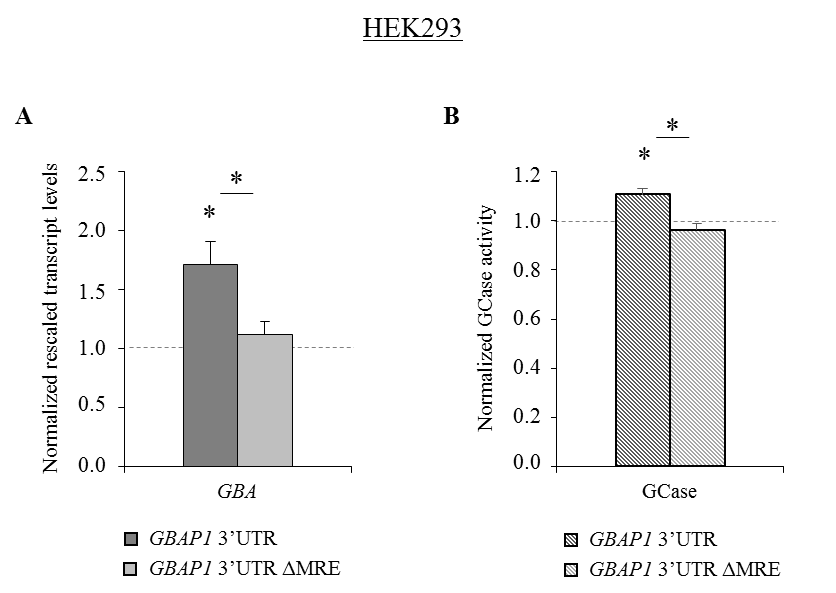


**Supplementary figure 3: *GBAP1* acts as a ceRNA titrating miR-22-3p and up-regulating *GBA* in HEK293 cells.**

**A)** The psiCHECK2 vectors, containing the 3’UTR region of *GBAP1* (with or without the miR-22-3p recognition element, ΔMRE) downstream of the luciferase reporter gene, were independently transfected in HEK293 cells together with the plasmid expressing the pre-miR-22-3p transcript. 24 hours after transfections, cells were collected for extracting total RNA for measurements of *GBA* endogenous levels by semi-quantitative real-time RT-PCRs. The value measured in cells transfected with an empty vector (psiCHECK2, mock) was set as 1 (dotted line).

**B)** Over-expression experiments of psiCHECK2 vectors, containing the two different 3’UTR regions of *GBAP1*, upon miR-22-3p over-expression were repeated for preparing protein lysates for endogenous GCase activity measurements. In this case, the GCase activity was measured 48 hours after transfections.

In all panels, error bars represent means +SEM of three biological replicates, each performed in triplicate. Significance levels of t-tests are shown. *P<0.05.


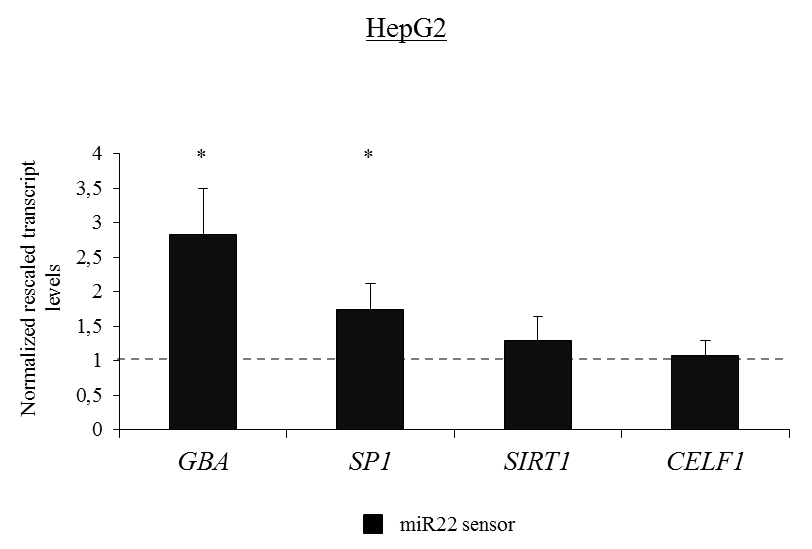


**Supplementary figure 4: miR-22-3p targets are efficiently titrated by an “artificial” sponge (miR-22 sensor).**

Effect of miR-22 sensor over-expression on the endogenous transcript levels of miR-22-3p targets in HepG2 cells. 24 hours after transfections, cells were collected for extracting total RNA for measurements by semi-quantitative real-time RT-PCRs of: i) *GBA*; ii) *SP1* (Sp1 Transcription Factor; known miR-22-3p target, positive control); iii) *SIRT1* (Sirtuin 1; known miR-22-3p target, positive control); and iv) *CELF1* (CUGBP, Elav-Like Family Member 1; negative control). The value measured in cells transfected with an empty vector (psiCHECK2, mock) was set as 1 (dotted line).

Error bars represent means +SEM of 3 independent biological replicates, each performed at least in triplicate. Significance levels of t-tests are shown. *P<0.05.


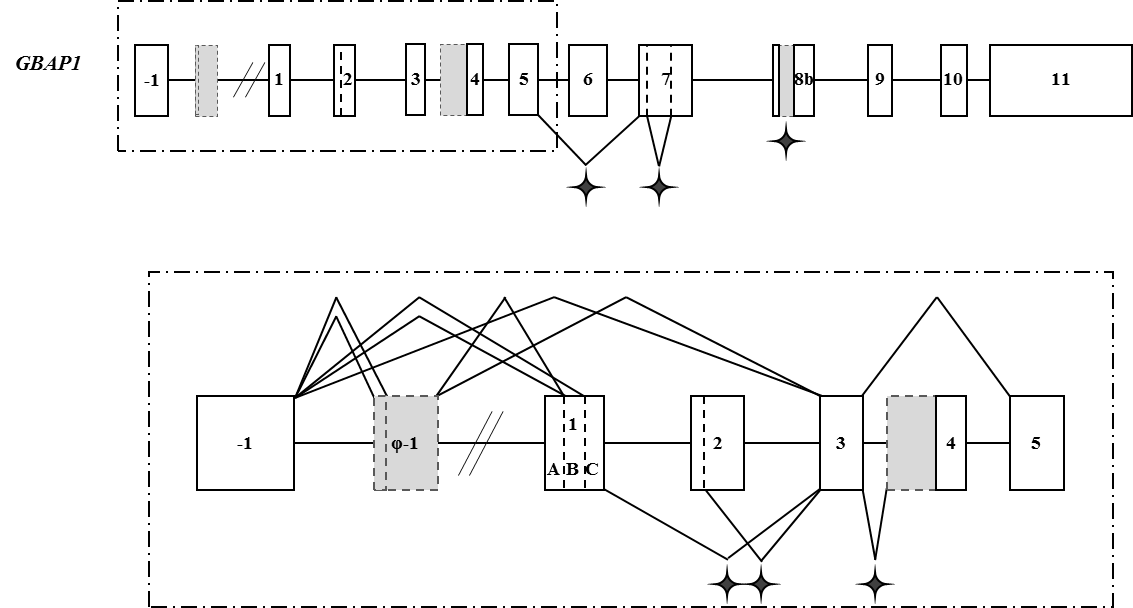


**Supplementary figure 5: *GBAP1* is characterized by multiple in-frame and out-of-frame splicing isoforms.**

Alternative splicing of the *GBAP1* pre-mRNA was analyzed in HepG2 cells, treated with the NMD inhibitor cycloheximide, by RT-PCR using primer couples specific for the pseudogene. In the upper part of the figure, a schematic representation of the whole gene is presented, with white boxes representing annotated exons, lines indicating introns, and broken lines pointing to the major splicing events characterizing the 3’ portion of the transcript. Grey boxes indicate the presence of additional exons or extensions of annotated exons. In the lower part of the figure, a magnification of the 5’ portion of the gene is reported, with all the identified alternative splicing events. Out-of-frame splicings leading to the introduction of a PTC are indicated in all cases by a star. The precise mapping of all identified splicing events was confirmed by Sanger sequencing of the relevant RT-PCR fragment.


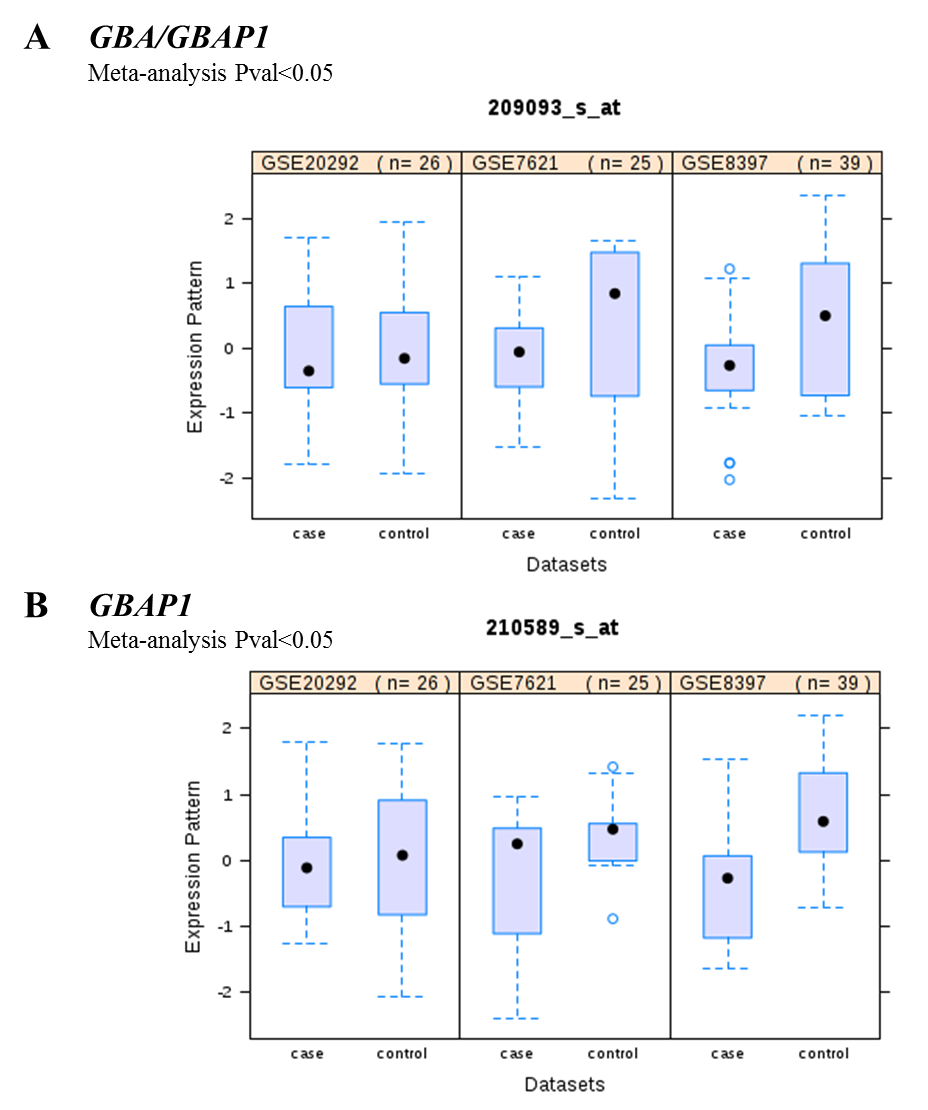


**Supplementary figure 6: *GBA* and *GBAP1* appear dowregulated in the *substantia nigra* of Parkinson’s disease patients.**

The figure shows box-plots comparing the relative gene expression of *GBA*/*GBAP1* (probe set 209093_s_at, hybridizing against both genes; **panel A**) and *GBAP1* (probe set 210589_s_at, specific for *GBAP1*; **panel B**) in the *substantia nigra* of Parkinson’s disease patients (case) and healthy controls (control). Both panels represent screen shots obtained from the INMEX program (Xia *et al.*, 2013), which rescales all expression levels in order to make data immediately comparable at visual inspection. For each box plot, the relevant dataset is indicated, together with the number of analyzed individuals. In the case of dataset GSE20292, three Parkinson’s disease patients were excluded from the analysis, since they resulted outliers (abnormal low levels of both *GBA* and *GBAP1*). The meta-analysis P values for the two analyzed probe sets is indicated.

**Supplementary table 1: MiRNAs predicted as potential targets of *GBA* and *GBAP1*.**

| ***miRNA***  ***(chromosomal position)**** | ***miRNA:GBA pairing***** | ***Brain expression****** | ***Literature data on neurodegenerative diseases*** |
| --- | --- | --- | --- |
| **miR-22-3p**  (chr17:1,617,197-1,617,281) | | 3' ugucaagaagUUGACCGUCGAa 5' miR-22 | | --- | | || |||||||| | | 5' cagccaggaaAAAUGGCAGCUc 3' *GBA* | | Cerebellum (643)  frontal cortex (219) | AD: Cheng *et al.*, 2013  ALS: Parisi *et al.*, 2013  HD: Jovicic *et al.*, 2013; Lee *et al.*, 2011  PD: Margis *et al.*, 2011 |
| **miR-132**  (chr17:1,953,202-1,953,302) | | 3' gcuGGUACCGACAUCUGACAAu 5' miR-132 | | --- | | ||| ||| |||||||| | | 5' ggcCCAAAACUGGAGACUGUUu 3' *GBA* | | Cerebellum (203)  frontal cortex (818) | AD: Smith *et al.*, 2015  ALS: Freischmidt *et al.*, 2013  HD: Lee *et al.*, 2011  PD: Alieva *et al.*, 2015 |
| **miR-212**  (chr17:1,953,565-1,953,674) | | 3' ccGGCACUGACCUCUGACAAu 5' miR-212 | | --- | | || ||||||||||||| | | 5' gcCCAAAACUGGAGACUGUUu 3' *GBA* | | Cerebellum (22)  frontal cortex (70) | AD: Smith *et al.*, 2015 |

Predictions were performed using microRNA.org, MicroCosm Targets, PITA, as well as the miRWalk2 suite (among the programs implemented in miRWalk2, we selected miRWalk2.0, RNA22, miRanda, miRDB, TargetScan, and PICTAR2). In all cases, default parameters were used.).

* According to UCSC genome browser (http://genome-euro.ucsc.edu/index.html) on Human Feb. 2009 (GRCh37/hg19) Assembly.

** The RNAhybrid software (http://bibiserv.techfak.uni-bielefeld.de/rnahybrid/) was used to visualize the miRNA:mRNA interactions.

*** According to deep-sequencing data available through miRBase (http://www.mirbase.org/); numbers in brackets refers to read counts per million of RNA-seq experiments (annotation confidence: high for miR-22 and miR-132; not reported for miR-212).

ALS: amyotrophic lateral sclerosis; AD: Alzheimer disease; HD: Huntington’s disease; PD: Parkinson’s disease.

**Supplementary table 2: Characteristics of Parkinson’s disease-related datasets included in the study.**

| ***Dataset***  ***(accession number)*** | ***Array type*** | ***PD cases / controls*** | ***Origin*** | ***Age at death***  ***(years ± SD)*** | ***Notes on PD patients*** | ***Tissue*** | ***Reference*** |
| --- | --- | --- | --- | --- | --- | --- | --- |
| GSE7621 | Affymetrix Human Genome U133 Plus 2.0 Array | 16 / 9 | Caucasian | Mean age cases: 77.9 ± 13.1  Mean age controls: n.a. | All PD subjects had advanced disease with a mean H&Y stage of 4.5 ± 0.7. | SN tissue from post-mortem brains | Lesnick *et al.*, 2007  Papapetropoulos *et al.*, 2006 |
| GSE8397 | Affymetrix Human Genome U133A Array | 24 / 15 | n.a. | Mean age cases: 80 ± 5.7  Mean age controls: 70.6 ± 12.5 | PD patients showed a mean disease duration of 13.4 ± 8.3 | SN tissue from post-mortem brains (NS split into medial and lateral portions) | Moran *et al.*, 2006 |
| GSE20292 | Affymetrix Human Genome U133A Array | 11 / 18 | n.a. | Mean age cases: 76.7  ±  6.2  Mean age controls: 71.2  ±  11.1 | - | SN tissue from post-mortem brains | Zhang *et al.*, 2005 |

Abbreviations: H&Y, Hoehn and Yahr scale; n.a., not available; PD, Parkinson’s disease; SD, standard deviation; SN, *substantia nigra*.

**Supplementary table 3: Primers used for cloning, mutagenesis, as well as expression profiling experiments.**

| ***Primer*** | ***Sequence (5’-3’) ^*^*** | ***Localization ***** | ***Application*** |
| --- | --- | --- | --- |
| *GBA*_ex9_F  *GBA*_ex10_R | ATTGGGTGCGTAACTTTGTC  TCCAGGTCGTTCTTCTGACT | exon 9, chr1:155,205,549-155,205,568  exon 10, chr1:155,205,040-155,205,059 | real-time RT-PCR assay to evaluate *GBA* expression |
| *GBAP1*_F  *GBAP1*_R | GGACCGACTGGAACCCAT  TCCAGGTCGTTCTTCTGACTG | exon 9, chr1:155,184,911-155,184,928  exon 10, chr1:155,184,413-155,184,433 | real-time RT-PCR assay to evaluate *GBAP1* expression |
| *SP1*_F  *SP1*_R | AGACAGTGAAGGAAGGGGCT  GCGTTTCCCACAGTATGACC | exon 4/5, chr12:53,800,523-53,803,150  exon 5, chr12:53,803,280-53,803,299 | real-time RT-PCR assay to evaluate *SP1* expression |
| *SIRT1*_F  *SIRT1*_R | TGTTATTGGGTCTTCCCTCAA  AAATGCAGATGAGGCAAAGG | exon 7, chr10:69,669,153-69,669,173  exon 8, chr10:69,672,275-69,672,294 | real-time RT-PCR assay to evaluate *SIRT1* expression |
| *CELF1*_F  *CELF1*_R | GAAGCCAGAAGGAAGGTCCA  TCCCAAAGGGCATAAACATC | exon 10/11, chr11:47,494,779-47,496,902  exon 11, chr11:47,494,697-47,494,716 | real-time RT-PCR assay to evaluate *CELF1* expression |
| *HMBS*_F  *HMBS*_R | GTTCAGGAGTATTCGGGGAAACC  TTCCTCAGGGTGCAGGATCTG | exon 8/9, chr11:118,960,963-118,962,132  exon 10, chr11:118,962,832-118,962,852 | reference gene for real-time RT-PCR assays |
| CX32_F  CX32_R | GCAGCAGCAGCCAGGTGTGG  ATACTCGGCCAATGGCAGTA | exon 1, chrX:70,435,108-70,435,127  exon 2, chrX:70,443,608-70,443,627 | reference gene for NMD experiments |
| CX43_F  CX43_R | AAAGTACCAAACAGCAGCGG  CTCCAGCAGTTGAGTAGGCT | exon 1, chr6:121,756,846-121,756,865  exon 2, chr6:121,768,038-121,768,057 | reference gene for NMD experiments |
| *PRKCA*_ex3*_F  *PRKCA*_4/5_R | TCCCCTGTATTGCTAGTCTGC  CGCAGGTGTCACATTTCATC | exon 3*, chr17:64,550,643-64,550,663  exon 4/5, chr17:64,637,571- 64,641,506 | positive control for NMD experiments |
| *PRKCA*_ex3/4F3  *PRKCA*_4/5_R | GGACCCGACACTGATGACC  CGCAGGTGTCACATTTCATC | exon3/4 chr17:64,492,387- 64,637,476  exon 4/5, chr17:64,637,571- 64,641,506 | negative control for NMD experiments |
| miR-22-3p_F1  Uni_R1 | GCTGCCAGTTGAAGAACT  CTCAGTCGCATAGCTTGAT | exon 3, chr17:1,617,210-1,617,227 | real-time RT-PCR assay to evaluate miR-22-3p expression |
| miR-132-3p_F  Uni_R1 | AGTCTACAGCCATGGTCG  CTCAGTCGCATAGCTTGAT | chr17:1,953,223-1,953,240 | real-time RT-PCR assay to evaluate miR-132-3p expression |
| miR-22_*Kpn*I_F  miR-22_*Xho*I_R | AGAGGTACCTTTCCCTTAGGAGCCTGT  GGCCTCGAGCAGCCCATTTCTGTCACCTT | chr17:1,617,301-1,617,320  chr17:1,617,133-1,617,152 | pre-miR-22 molecular cloning |
| miR-132_*Kpn*I_F  miR-132_*Xho*I_R | AGAGGTACCCAGTCCCCGTCCCTCAG  GGCCTCGAGCACGTGGGATCTTGACTCG | chr17:1,953,507-1,953,523  chr17:1,953,123-1,953,141 | pre-miR-132 molecular cloning |
| UTR_*GBA*-*GBAP1*_*Sgf*I_F  UTR_*GBA*-*GBAP1*_*Not*I_R | ACCGGCGATCGCTCACCTGGCTACTCCATTCA  AAGGAAAAAGCGGCCGCCCACCCAGAATAAAGCCACT | exon 11, chr1:155,204,811-155,204,830  chr1:155,204,214-155,204,233 | *GBA* and *GBAP1* 3’UTR molecular cloning |
| psiUx_F  psiUx_R | GATCTTCCCCATCGGTGAT  CGCTGATCGGAAGTGAGAAT | psiUx  psiUx | recombinant bacterial colony screening |
| psiCHECK_2_F  psiCHECK_2_R | AGGACGCTCCAGATGAAATG  AGGACGCTCCAGATGAAATG | psiCHECK-2  psiCHECK-2 | recombinant bacterial colony screening |
| 3’UTR_*GBAP1*_ΔMRE_F  3’UTR_*GBAP1*_ΔMRE_R | TCCTATGGCACCAGCCAGGAAAAATCTTAAAGGAGAAAATGTTTGAGCCC  GGGCTCAAACATTTTCTCCTTTAAGATTTTTCCTGGCTGGTGCCATAGGA |  | miR-22 MRE site-directed mutagenesis (deletion) |
| *GBAP1*_EX-1_F | GGGCTGCTTCTTGACTTCC | exon -1, chr1:155,197,281-155,197,299 | RT-PCR assay to characterize *GBAP1* splicing pattern |
| *GBAP1*_EX-1/-φ1_F | TTCTCTTCGCCGACGGTT | exon -1/intron 1, chr1:155,197,168-155,194,848 | RT-PCR assay to characterize *GBAP1* splicing pattern |
| *GBAP1*_-28-φ1_F | TGACAGGGCTTTCCCTATGT | intron 1, chr1:155,194,858-155,194,877 | RT-PCR assay to characterize *GBAP1* splicing pattern |
| *GBAP1*_-φ1_F | CTGGGCTCAAAAGATCCTCA | intron 1, chr1:155,194,816-155,194,835 | RT-PCR assay to characterize *GBAP1* splicing pattern |
| *GBAP1*_EX-1/1_F | CTCTTCGCCGACGTGGA | exon -1/1, chr1:155,197,168-155,188,730 | RT-PCR assay to characterize *GBAP1* splicing pattern |
| *GBAP1*_EX-1/1B_F | CTCTTCGCCGACGAGACT | exon -1/1, chr1:155,197,168-155,188,711 | RT-PCR assay to characterize *GBAP1* splicing pattern |
| *GBAP1*_EX-1/1C_F | TCTTCGCCGACGTGACC | exon -1/1, chr1:155,197,168-155,188,674 | RT-PCR assay to characterize *GBAP1* splicing pattern |
| *GBAP1*_EX-1/3_F | CTCTTCGCCGACGGTGC | exon -1/1, chr1:155,197,168-155,187,837 | RT-PCR assay to characterize *GBAP1* splicing pattern |
| *GBAP1*_EX2A_F | TCCCAAGCCTTCGGGTAG | exon 2, chr1:155,188,248-155,188,265 | RT-PCR assay to characterize *GBAP1* splicing pattern |
| *GBAP1*_EX2_F | TTCGGGTAGGGTAAGCATCA | exon 2, chr1:155,188,237-155,188,256 | RT-PCR assay to characterize *GBAP1* splicing pattern |
| *GBAP1*_EX2_R | CCACGACACTGCCTGAAGTA | exon 2, chr1:155,188,190-155,188,209 | RT-PCR assay to characterize *GBAP1* splicing pattern |
| *GBAP1*_EX3_R | CCGGTGCAATTAGCCTGTAT | exon 3, chr1:155,187,760-155,187,779 | RT-PCR assay to characterize *GBAP1* splicing pattern |
| *GBAP1*_EX3/5_F | TGCACCGGCACAGGAAT | exon 3/5, chr1:155,187,161-155,187,767 | RT-PCR assay to characterize *GBAP1* splicing pattern |
| *GBAP1*_EX3/5_R | GATGTTATATCCGATTCCTGTGC | exon 3/5, chr1:155,187,148-155,187,766 | RT-PCR assay to characterize *GBAP1* splicing pattern |
| *GBAP1*_EX4_F | TAAGATTTCGCCGCCTATCA | exon 4, chr1:155,187,360-155,187,379 | RT-PCR assay to characterize *GBAP1* splicing pattern |
| *GBAP1*_EX4_R | GAGCCTGAGTCCGTAGCAGT | exon 4, chr1:155,187,333-155,187,352 | RT-PCR assay to characterize *GBAP1* splicing pattern |
| *GBAP1*_EX5_R | ATAGGTGTAGGTGCGGATGG | exon 5, chr1:155,187,097-155,187,116 | RT-PCR assay to characterize *GBAP1* splicing pattern |
| *GBAP1*_EX7_F | TGAGTGGATACCCCTTCCAG | exon 7, chr1:155,186,316-155,186,335 | RT-PCR assay to characterize *GBAP1* splicing pattern |
| *GBAP1*_IVS8A_R | GGGAACAGGTGGTGTGTCTC | exon 8, chr1:155,185,466-155,185,485 | RT-PCR assay to characterize *GBAP1* splicing pattern |
| *GBAP1*_EX8A/8B_R | ACCCACACAGGCCTTTAGC | exon 8A/8B, chr1:155,185,429-155,185,557 | RT-PCR assay to characterize *GBAP1* splicing pattern |
| *GBAP1*_F | GGACCGACTGGAACCCAT | exon 9, chr1:155,184,911-155,184,928 | RT-PCR assay to characterize *GBAP1* splicing pattern |
| *GBAP1*_EX9_R | ATGTCTACAATGATGGGTTCCAG | exon 9, chr1:155,184,899-155,184,921 | RT-PCR assay to characterize *GBAP1* splicing pattern |
| *GBAP1*_EX11_R | TGAATGGAGTAGCCAGGTGA | exon 11, chr1:155,184,184-155,184,203 | RT-PCR assay to characterize *GBAP1* splicing pattern |

* Underlined sequences corresponds to nucleotides added at the primer end to introduce a site for restriction digestion in cloning experiments

** According to UCSC genome browser (http://genome-euro.ucsc.edu/index.html) on Human Feb. 2009 (GRCh37/hg19) Assembly.

**SUPPLEMENTARY REFERENCES**

Alieva AKh, Filatova EV, Karabanov AV, Illarioshkin SN, Limborska SA, Shadrina MI, Slominsky PA. miRNA expression is highly sensitive to a drug therapy in Parkinson's disease. Parkinsonism Relat Disord 2015; 21: 72**−**4.

Barrett T, Troup DB, Wilhite SE, Ledoux P, Rudnev D, Evangelista C, Kim IF, Soboleva A, Tomashevsky M, Marshall KA, Phillippy KH, Sherman PM, Muertter RN, Edgar R. NCBI GEO: archive for high-throughput functional genomic data. Nucleic Acids Res 2009; 37(Database issue): D885**−**90.

Barrett T, Wilhite SE, Ledoux P, Evangelista C, Kim IF, Tomashevsky M, Marshall KA, Phillippy KH, Sherman PM, Holko M, Yefanov A, Lee H, Zhang N, Robertson CL, Serova N, Davis S, Soboleva A. NCBI GEO: archive for functional genomics data sets-update. Nucleic Acids Res 2013; 41(Database issue): D991**−**5.

Cheng XR, Cui XL, Zheng Y, Zhang GR, Li P, Huang H, Zhao YY, Bo XC, Wang SQ, Zhou WX, Zhang YX. Nodes and biological processes identified on the basis of network analysis in the brain of the senescence accelerated mice as an Alzheimer's disease animal model. Front Aging Neurosci 2013; 5: 65.

Freischmidt A, Müller K, Ludolph AC, Weishaupt JH. Systemic dysregulation of TDP-43 binding microRNAs in amyotrophic lateral sclerosis. Acta Neuropathol Commun 2013; 1: 42.

Gentleman RC, Carey VJ, Bates DM, Bolstad B, Dettling M, Dudoit S, Ellis B, Gautier L, Ge Y, Gentry J, Hornik K, Hothorn T, Huber W, Iacus S, Irizarry R, Leisch F, Li C, Maechler M, Rossini AJ, Sawitzki G, Smith C, Smyth G, Tierney L, Yang JY, Zhang J. Bioconductor: open software development for computational biology and bioinformatics. Genome Biol 2004; 5: R80.

Jovicic A, Zaldivar Jolissaint JF, Moser R, Silva Santos Mde F, Luthi-Carter R. MicroRNA-22 (miR-22) overexpression is neuroprotective via general anti-apoptotic effects and may also target specific Huntington's disease-related mechanisms. PLoS One 2013; 8: e54222.

Lee ST, Chu K, Im WS, Yoon HJ, Im JY, Park JE, Park KH, Jung KH, Lee SK, Kim M, Roh JK. Altered microRNA regulation in Huntington's disease models. Exp Neurol 2011; 227: 172**−**9.

Lesnick TG, Papapetropoulos S, Mash DC, Ffrench-Mullen J, Shehadeh L, de Andrade M, Henley JR, Rocca WA, Ahlskog JE, Maraganore DM. A genomic pathway approach to a complex disease: axon guidance and Parkinson disease. PLoS Genet. 2007; 3: e98.

Margis R, Margis R, Rieder CR. Identification of blood microRNAs associated to Parkinson’s disease. J Biotechnol 2011; 152: 96**−**101.

Moran LB, Duke DC, Deprez M, Dexter DT et al. Whole genome expression profiling of the medial and lateral substantia nigra in Parkinson's disease. Neurogenetics 2006; 7: 1**−**11.

Papapetropoulos S, Ffrench-Mullen J, McCorquodale D, Qin Y, Pablo J, Mash DC. Multiregional gene expression profiling identifies MRPS6 as a possible candidate gene for Parkinson's disease. Gene Expr. 2006; 13: 205**−**15.

Parisi C, Arisi I, D'Ambrosi N, Storti AE, Brandi R, D'Onofrio M, Volonté C. Dysregulated microRNAs in amyotrophic lateral sclerosis microglia modulate genes linked to neuroinflammation. Cell Death Dis 2013;4:e959.

R Core Team. R: A language and environment for statistical computing. R Foundation for Statistical Computing, Vienna, Austria. 2013. URL http://www.R-project.org/.

Smith PY, Hernandez-Rapp J, Jolivette F, Lecours C, Bisht K, Goupil C, Dorval V, Parsi S, Morin F, Planel E, Bennett DA, Fernandez-Gomez FJ, Sergeant N, Buée L, Tremblay MÈ, Calon F, Hébert SS. miR-132/212 deficiency impairs tau metabolism and promotes pathological aggregation in vivo. Hum Mol Genet 2015; 24: 6721**−**35.

Xia J, Fjell CD, Mayer ML, Pena OM, Wishart DS, Hancock RE. INMEX-a web-based tool for integrative meta-analysis of expression data. Nucleic Acids Res 2013; 41(Web Server issue): W63**−**70.

Zhang Y, James M, Middleton FA, Davis RL. Transcriptional analysis of multiple brain regions in Parkinson's disease supports the involvement of specific protein processing, energy metabolism, and signaling pathways, and suggests novel disease mechanisms. Am J Med Genet B Neuropsychiatr Genet 2005; 137B: 5**−**16.
